# Podoconiosis, trachomatous trichiasis and cataract in northern Ethiopia: A comparative cross sectional study

**DOI:** 10.1371/journal.pntd.0005388

**Published:** 2017-02-10

**Authors:** Helen Burn, Sintayehu Aweke, Tariku Wondie, Esmael Habtamu, Kebede Deribe, Saul Rajak, Stephen Bremner, Gail Davey

**Affiliations:** 1 Wellcome Trust Brighton and Sussex Centre for Global Health Research, Brighton and Sussex Medical School, Brighton, United Kingdom; 2 The Carter Center, Addis Ababa, Ethiopia; 3 International Centre for Eye Health, London School of Hygiene & Tropical Medicine, London, United Kingdom; 4 School of Public Health, Addis Ababa University, Addis Ababa, Ethiopia; 5 Sussex Eye Hospital, Brighton, Sussex, United Kingdom; 6 Brighton and Sussex Medical School, Brighton, United Kingdom; Texas A&M University College Station, UNITED STATES

## Abstract

**Background:**

Rural populations in low-income countries commonly suffer from the co-morbidity of neglected tropical diseases (NTDs). Podoconiosis, trachomatous trichiasis (both NTDs) and cataract are common causes of morbidity among subsistence farmers in the highlands of northern Ethiopia. We explored whether podoconiosis was associated with cataract or trachomatous trichiasis (TT) among this population.

**Methods:**

A comparative cross-sectional study was conducted in East Gojam region, Amhara, Ethiopia in May 2016. Data were collected from patients previously identified as having podoconiosis and from matched healthy neighbourhood controls. Information on socio-demographic factors, clinical factors and past medical history were collected by an interview-administered questionnaire. Clinical examination involved grading of podoconiosis by examination of both legs, measurement of visual acuity, direct ophthalmoscopy of dilated pupils to grade cataract, and eyelid and corneal examination to grade trachoma. Multiple logistic regression was conducted to estimate independent association and correlates of podoconiosis, TT and cataract.

**Findings:**

A total of 700 participants were included in this study; 350 podoconiosis patients and 350 healthy neighbourhood controls. The prevalence of TT was higher among podoconiosis patients than controls (65 (18.6%) vs 43 (12.3%)) with an adjusted odds ratio OR 1.57 (95% CI 1.02–2.40), p = 0.04. There was no significant difference in prevalence of cataract between the two populations with an adjusted OR 0.83 (95% CI 0.55–1.25), p = 0.36. Mean best visual acuity was 0.59 (SD 0.06) in podoconiosis cases compared to 0.44 (SD 0.04) in controls, p<0.001. The proportion of patients classified as blind was higher in podoconiosis cases compared with healthy controls; 5.6% vs 2.0%; adjusted OR 2.63 (1.08–6.39), P = 0.03.

**Conclusions:**

Individuals with podoconiosis have a higher burden of TT and worse visual acuity than their matched healthy neighbourhood controls. Further research into the environmental and biological reasons for this co-morbidity is required. A shared approach to managing these two NTDs within the same population could be beneficial.

## Author summary

Podoconiosis is an NTD causing chronic leg swelling in subsistence farming communities in the tropics. There is no research on the association between podoconiosis and two common causes of blindness and visual impairment; trachomatous trichiasis (TT) and cataract. TT is the blinding consequence of conjunctival scarring in trachoma, the leading infectious cause of blindness globally. Cataract is an age-related disease of the lens and remains the leading cause of visual impairment worldwide. Both podoconiosis and TT are NTDs endemic to Ethiopia and promote poverty through many factors such as disability, reduced economic productivity and stigma. This comparative cross-sectional study explored the association between podoconiosis and these two eye diseases. We found that podoconiosis patients were burdened with higher levels of blindness and low vision, had higher prevalence of TT and more severe cataract than their matched neighbourhood controls. These findings can help to direct an integrated approach to managing these two NTDs (podoconiosis and TT) and trigger further research in to the wider context of the double burden of eye disease and NTDs.

## Introduction

Neglected tropical diseases (NTDs) do not occur in isolation but have substantial geographical overlap. This results in an increased burden of co-morbidity within a population, commonly leading to individuals suffering from one or more NTDs[1]. These conditions share common risk factors including lack of access to clean water, sanitation and hygiene practices[2]. We describe here a study in which we aimed to explore the association between the NTD podoconiosis and two common eye diseases; one an NTD (trachomatous trichiasis) and another a common age-related disabling eye disease (cataract), within a rural population in northern Ethiopia.

Podoconiosis is a non-filarial elephantiasis that predominantly affects subsistent farmers in areas of red clay soil covered highlands of tropical Africa, Northern India and South and Central America [3,4,5,6]. It causes painful swelling and deformity of the lower legs with acute, painful inflammatory events known as acute adenolymphangioadenitis (ALA) [7]. Although the aetiology is not fully understood, current evidence suggests it occurs as a result of both a genetic susceptibility and exposure to irritant mineral particles in volcanic soils [7,8,9,10]. The disease carries a high socio-economic burden and is highly stigmatizing [11,12,13]. Nationwide mapping in 2015 found podoconiosis to be endemic in 345 districts in Ethiopia with a prevalence of 4% [14]. East Gojam zone in Amhara region, where this study took place, has a podoconiosis prevalence of 3.3% [15]. The recommended management of podoconiosis is inexpensive and simple involving foot hygiene, emollient, bandaging, exercise and wearing socks and shoes [16].

Anecdotally clinicians and researchers working with populations affected by podoconiosis report a high prevalence of cataract and trachomatous trichiasis (TT) among affected individuals.

The 2006 National Blindness Survey of Ethiopia found the prevalence of blindness and low vision in Amhara to be 1.4% and 4.9%, respectively [19]Nationwide, cataract and trachomatous corneal opacity were found to be the leading causes of blindness with cataract accounting for 49.9% and trachomatous corneal opacity 11.5% [19].

Cataract is a clouding of the lens, which results in decreased vision. The leading cause is age. Cataract develops at a younger age in tropical and poor countries. The precise reasons for this are unclear but it is likely to be due to a combination of factors including episodes of dehydration in early life, diet, and solar and heat radiation [18].

Trachoma is the leading infectious cause of blindness worldwide [17]. The disease starts in childhood with recurrent infection of the tarsal conjunctiva by *Chlamydia trachomatis* producing chronic inflammation. This leads to tarsal scarring followed by entropion (inward rotation of the eyelid) and trichiasis, a painful condition where eyelashes rub on the cornea causing corneal scarring.

Ethiopia is one of the most trachoma-affected countries in the world; in 2016 nearly 50% of people at risk of trachoma globally live in Ethiopia, Malawi and Nigeria [20]. The number of people with TT awaiting surgery in 2016 is 693,000, again the largest in the world [20]. The 2006 survey found Amhara Regional State bears an estimated 45% of the national trichiasis burden with approximately one in twenty of all adults suffering from the condition. Since 2001 TT surgery has been provided by the Amhara Regional Trachoma Control Programme with health workers throughout Amhara Region trained to perform TT surgery, including 40 in East Gojam Zone.

Ethiopia carries a high burden of Neglected Tropical Diseases, and is estimated to have the highest burden of podoconiosis and trachoma in Sub-Saharan Africa [21]. Both podoconiosis and trachoma are part of the Ethiopian Government’s National Neglected Tropical Disease (NTD) Master Plan [22]. Launched in 2013, the NTD Master Plan pledged to achieve WHO NTD elimination and control targets by 2020. Evidence of the presence of the double burden of podoconiosis and TT will facilitate decisions on the integration of policy and treatment programmes for these two NTDs.

## Methods

### Study setting and design

A comparative cross-sectional study was carried out in East Gojam Zone, Amhara Region, Ethiopia during four weeks in May 2016. Amhara is one of 9 regions and 2 city administrations of Ethiopia and is divided into 10 zones. East Gojam zone is divided into 20 *woredas* (equivalent of districts), which are divided into *kebeles* (the lowest governmental administrative unit). Within East Gojam Zone the *Woreda* Enarj Enawga was selected for this study as it was known to have a high burden of podoconiosis and access to recent complete data from the Podoconiosis Burden Assessment 2015 [23]. Enarj Enawga has a population of 167,402, of which 92% are rural inhabitants [24]. A non-random convenience sampling method was used to select 12 *kebeles* within Enarj Enwarga based on geographical location and accessibility.

### Sample size

Assuming the prevalence of cataract in healthy controls is 8% and the effect estimate is an odds ratio of 2, we calculated the need to recruit 336 cases and 336 cases for 80% power and 5% significance [19].

### Study population and sampling procedure

Cases were located using the Podoconiosis Burden Assessment 2015 which provided participant name, household name, and village for all individuals over the age of 40 with podoconiosis within each *kebele* [23]. Every podoconiosis case over age 40 within each of the 12 *kebeles* was invited to take part in the study. In total, 460 podoconiosis cases were identified. They were invited to participate in the study through both verbal information via the *Kebele* Leader and a written letter of invitation asking them to attend a health centre on a particular date.

Neighbourhood controls matched by age (+/- 5 years), sex and village were selected randomly from a residents’ register detailing each individual living within each village. Village leaders, Health Extension Workers and *kebele* leaders were able to locate each control selected. All were provided with information about the study and invited by letter to attend a health centre within their *kebele* on a particular date. Controls were excluded if they were under 40 years old, had podoconiosis on clinical examination, or had a first-degree relative with podoconiosis.

### Measurement and data collection

The primary outcomes for participants with podoconiosis and healthy neighbourhood controls were: 1) presence of cataract 2) presence of TT.

Secondary outcomes were: grade of cataract, severity of TT, visual acuity, socio-demographic variables (age, sex, occupation), socio-economic status, history of hypertension, history of diabetes, previous eye surgery, previous eye trauma, previous eye diagnosis, number of acute podoconiosis attacks and stage of podoconiosis.

Data were collected through an interview-administered questionnaire followed by clinical examination. Questionnaires were administered by trained Amharic-speaking data collectors. Visual acuity was tested using PEEK Visual Acuity on smartphones recorded on a LogMar scale [25]. LogMAR values were categorised according to the ICD-10 classification of visual impairment; normal vision, ≤ 0.4; mild impairment, 0.4–1.0; severe impairment, 1.0–1.3; blindness ≥1.3 [26]. In this study we present only best unaided vision as no patients had spectacles for distance correction. Podoconiosis grading involved examination of both legs using a validated 5 stage grading system carried out by a local Podoconiosis Nurse Specialist [27]. An ophthalmic officer and a medical doctor carried out the clinical eye examinations. Tropicamide 1.0% mydriatic drops were administered to both eyes. Cataract was examined using a direct ophthalmoscope at 30cm from dilated pupils as slit lamp examination was unavailable. Cataract was classified into five grades according to Mehra and Minassian’s method of grading in eye surveys using degree of opacity in the red reflex to define the grade [28]. Grades 4 and 5 were regarded as severe matured cataract requiring surgery. Both eyes were then examined using a torch and x2.5 magnifying binocular loop for signs of trachoma. Each eye was examined for in-turned lashes (TT), the cornea inspected for central corneal opacities (opacities within central 4mm) and the upper conjunctiva everted and examined for inflammation (Trachomatous Inflammation—Follicular (TF) and Trachomatous Inflammation—Intense (TI)) and scarring (Trachomatous Scarring (TS)). The WHO simplified trachoma grading system was used to define each of these stages [29]. TT was defined as one or more lashes touching the globe or evidence of eyelash epilation [17]. Severity of TT was recorded by counting the number of lashes touching the globe when looking straight ahead and subdivided into corneal lashes (touching cornea) or peripheral lashes (touching medial or lateral conjunctiva). TT severity was classified into two groups: major as >5 peripheral or corneal lashes and minor as <6 peripheral or corneal lashes. Patients were told the findings of the eye examination at the end of the study. Individuals with signs of active trachoma (TF and/or TI) were offered treatment with 1% tetracycline eye ointment. TT patients were referred to health centres where free TT surgery was available. Patients with grade 4 or 5 cataract were referred to Debre Markos Hospital for cataract surgery. All podoconiosis patients were counseled by a Podoconiosis Specialist Nurse for podoconiosis management and enrolled into existing podoconiosis clinics.

### Statistical analysis

Data were coded, entered, cleaned and analysed using IBM SPSS Statistics version 22. Descriptive analysis of the socio-demographic and clinical characteristics of cases and controls was performed. When comparing simple frequencies, the χ^2^ test was used to establish significance. Means were compared using the independent t-test. Principal Components Analysis was used to reduce 15 wealth index factors (gained from the interview-administered questionnaire) into three household index values taking the first component as a measure of economic status divided into three categories: poor, middle class and wealthy [30,31]. A logistic regression model was used to determine the clinical and socio-demographic correlates of trachomatous trichiasis and cataract. The model was used to measure the association between podoconiosis and these two eye diseases (cataract and TT) adjusting for sex, age, occupation and socio-economic status. These confounding factors were chosen prior to data collection. They were chosen as factors likely to influence the association between podoconiosis and these two eye diseases based on previous literature [32,33,34,35,42].

### Ethical considerations

Ethical approval was gained from Amhara Regional Health Bureau and the Research Governance & Ethics Committee of Brighton & Sussex Medical School. An Amharic-speaking study supervisor gave, to each of the participants, an introduction to the study and the reasons why it was being conducted. Then, all participants were given written information in Amharic outlining the reason for the study and what would be involved if they chose to participate. If participants were unable to read or write, the information sheet was read to them individually. Informed consent was gained by signature and a thumbprint was used if the participant was unable to write [36]. The consent was then countersigned by an independent witness. Study participants identified with ocular disease or podoconiosis were managed as per local protocol. A podoconiosis nurse was present throughout the study to provide education regarding podoconiosis treatment and integration into existing clinics.

## Results

### Socio-demographic characteristics

A total of 700 participants were included in this study: 350 podoconiosis cases and 350 healthy neighbourhood controls. The socio-demographic characteristics of cases and controls are described in Table 1. More cases (60.3%) and controls (62.6%) were male than female. The mean age distribution between cases and controls was similar at 57 and 56 years respectively. The great majority of both cases and controls were rural farmers and married. However, compared to their neighbourhood controls, significantly larger numbers of podoconiosis cases were either divorced or widowed; 102 (29.2%) vs 56 (16.0%), p = 0.001, and lived in poorer households; 146 (41.7%) vs 88 (25.1%); p<0.001.

**Table 1:** Socio-demographic comparisons between cases and controls.

Variable		Podoconiosis cases n = 350 (%)	Healthy neighbourhood controls n = 350 (%)	P value*
**Gender**	*Male*	211 (60.3)	219 (62.6)	0.51
	*Female*	138 (39.4)	131 (37.4)	
**Age**	*Mean (SD)*	57 (12.0)	56 (11.1)	0.16**
	*40–50*	126 (36.0)	130 (37.1)	0.31
	*51–60*	105 (30.0)	104 (29.7)	
	*61–70*	67 (19.1)	81 (23.1)	
	*71–80*	45 (12.9)	31 (8.9)	
	*81–90*	7 (2.0)	4 (1.1)	
**Occupation**	*Farmer*	335 (95.7)	345 (98.6)	0.18
	*Other*	15 (4.2)	5 (1.4)	
**Marital Status**	*Single*	7 (2.0)	3 (0.9)	<0.001
	*Married*	241 (68.9)	291 (83.1)	
	*Divorced*	45 (12.9)	23 (6.6)	
	*Widowed*	57 (16.3)	33 (9.4)	
Socio-economic status***	*Poor*	146 (41.7)	88 (25.1)	<0.001
	*Moderate*	113 (32.3)	127 (36.3)	
	*Wealthy*	91 (26.0)	135 (38.6)	
**Distance travelled to water (mins)**	, *<5*	257 (73.4)	277 (79.1)	0.19
	*5–10*	47 (13.4)	41 (11.7)	
	*10–20*	28 (8.0)	23 (6.6)	
	*>20*	18 (5.1)	9 (2.6)	

*P value was calculated using Chi-squared test.

**P value was calculated using t test to compare difference in means.

*** Calculated using Principal Component Analysis to reduce 15 wealth index variables down to three categories of socio-economic status.

### Clinical characteristics

Over half of all podoconiosis patients had a first-degree relative with podoconiosis and had experienced an acute attack in the past 30 days. The median stage of podoconiosis for both legs was stage 2 (defined as persistent below knee swelling) [27]. The clinical characteristics of cases and controls are described in Table 2. Few patients in either group had a history of hypertension and diabetes. Compared to the controls, a significantly higher proportion of podoconiosis cases had an ocular problem; 52 (14.9%) vs 86 (24.6%), p = 0.001, and had had eye surgery; 49 (14.0%) vs 75 (21.4%); p = 0.01. In particular, compared to controls, a higher proportion of podoconiosis cases had had TT surgery (61 (17.4%) in cases vs 42 (12.0%) in controls, p = 0.04), a diagnosis of TT (62 (17.7%) in cases vs 40 (11.4%) in controls, p = 0.03) or a diagnosis of cataract (16 (4.6%) in cases vs 6(1.7%) in controls, p = 0.02).

**Table 2:** Clinical variables comparison between podoconiosis cases and controls.

			Podoconiosis cases n = 350 (%)	Healthy neighbourhood controls n = 350 (%)	P value*
**Diabetes**	*No*		345 (98.6)	347 (99.1)	0.60
	*Yes*		4 (1.1)	3 (0.9)	
**Hypertension**	*No*		336 (96.0)	345 (98.6)	0.04
	*Yes*		14 (4.0)	5 (1.4)	
**Previous eye surgery**	*No*		275 (78.6)	301 (86.0)	0.01
	*Yes*		75 (21.4)	49 (14.0)	
		*TTS*	61 (17.4)	42 (12.0)	0.04
		*Cataract*	9 (2.6)	5 (1.4)	0.28
		*Both*	4 (1.1)	2 (0.6)	0.40
		*Other*	0 (0.0)	0 (0.0)	
**Ocular problem**	*No*		264 (75.4)	298 (52.0)	0.001
	*Yes*		86 (24.6)	52 (14.9)	
	*Yes*	*TT and Cataract*	6 (1.7)	5 (1.4)	0.76
		*Cataract*	16 (4.6)	6 (1.7)	0.03
		*TT*	62 (17.7)	40 (11.4)	0.02
		*Glaucoma*	0 (0.0)	1 (0.3)	
		*Refractive*	1 (0.3)	0 (0)	
		*Corneal Opacity*	1 (0.3)	0 (0)	
		*Other*	0 (0)	0 (0)	

*P value was calculated using Chi-squared test.

Podoconiosis patients were found to have significantly lower visual acuity than healthy controls. (Table 3). Mean best visual acuity was 0.59 (SD 0.06) in podoconiosis cases compared to 0.44 (SD 0.04) in controls, p<0.001. The proportion of patients classified as blind was significantly higher in the podoconiosis group; 5.6% vs 2.0%; OR 2.97 (95% CI 1.24–7.11), p = 0.02. When adjusted for age, sex and socioeconomic status the association remained significant; adjusted OR 2.63 (1.08–6.39), p = 0.03.

**Table 3:** Clinical and socio-demographic correlates of blindness in East Gojam, Ethiopia.

Variables	Best Visual Acuity		Odds ratio	P value	Adjusted odds ratio*	P value
	*Not blind*^¶^	*Blind*^¶^				
***Podoconiosis status***						
**Patient**	331	19	2.97 (1.24–7.11)	0.02	2.63 (1.08–6.39)	0.03
**Healthy control**	343	7	1		1	
***Age***			0.94 (0.90–0.97)	0.001	0.93 (0.90–0.97)	<0.001
***Sex***						
**Female**	251	18	3.80 (1.63–8.85)	0.002	3.79 (1.48–9.69)	0.005
**Male**	423	8	1		1	
Wealth index**						
**Poor**	204	20	0.88 (0.20–0.38)	0.002	0.17 (0.04–0.78)	0.02
**Moderate**	238	4	0.52 (0.93–2.84)	0.51	0.62 (0.11–3.47)	0.56
**Wealthy**	231	3	1		1	
***Occupation***						
**Farmer**	653	25	1.24 (0.16–9.61)	0.83	1.29 (0.15–10.92)	0.82
**Other**	21	1	1		1	

^¶^Blindness defined according to ICD-10 classification of visual impairment based on best LogMAR visual acuity <1.3

*Binary logistic regression controlling for podoconiosis group, age, sex, occupation and socio-economic status

** Calculated using Principal Components Analysis to reduce 15 wealth index variables down to three categories of socio-economic status.

### TT and cataract prevalence among cases and controls

The prevalence of TT was higher in cases (65, 18.6%) than controls (43, 12.3%); OR 1.63; (95% CI 1.07–2.47), p = 0.02. 13 out of 65 (20%) cases had major trichiasis compared to 5 out of 43 (11%) of healthy controls (p = 0.16). The odds of having TT remain significantly greater for individuals with podoconiosis after adjustment for age, sex, occupation, socio-economic status and distance from water; adjusted OR 1.57 (95% CI 1.02–2.40), p = 0.04. Female patients had greater odds of TT compared to male patients OR 1.58 (95% CI 1.38–1.77), p<0.001 (Table 4).

**Table 4:** Clinical and socio-demographic correlates of any TT in East Gojam, Ethiopia.

Variables	Any TT n = 700		OR	P value	AOR*	P value
	Yes	No				
***Podoconiosis status***						
**Patient**	65	285	1.63 (1.07–2.47)	0.02	1.57 (1.02–2.40)	0.04
**Healthy control**	43	207	1		1	
***Age***			0.99 (0.96–1.01)	0.47	1.01 (0.90–1.03)	0.13
***Sex***						
**Male**	48	382	0.45 (0.30–0.70)	0.001	0.42 (0.27–0.66)	0.003
**Female**	60	210	1		1	
Wealth index**						
**Poor**	45	189	0.56 (0.33–0.93)	0.03	0.96 (0.61–1.61)	0.96
**Moderate**	31	209	1.04 (0.60–1.82)	0.88	0.82 (0.45–1.43)	0.81
**Wealthy**	32	194	1		1	
***Occupation***						
**Farmer**	104	576	0.55 (0.20–1.53)	0.26	0.90 (0.29–2.86)	0.86
**Other**	4	16	1		1	
***Distance from water***						
**<5 mins**	78	456	0.76 (0.47–1.22)	0.26	0.70 (0.44–1.12)	0.15
**>5 mins**	30	136	1		1	

* Binary logistic regression controlling for podoconiosis group, age, sex, occupation, socio-economic status and distance from water.

** Calculated using Principal Component Analysis to reduce 15 wealth index variables down to three categories of socio-economic status.

No significant difference in cataract prevalence was found between the two groups; 272 (77.7%) vs 286, (81.7%), OR, 0.87; (95% CI, 0.59–1.26), p = 0.47. However, podoconiosis cases were shown to have more severe cataract. The number of patients with grade 4 or 5 cataract in either eye or both was 37 (10.6%) for cases vs 29 (8.6%) for controls (p = 0.01). Many more eyes in the podoconiosis case group could not be examined due to severe corneal opacity or phthisis; 43 out 700 (6.1%) compared with 14 out of 700 (2%). The odds of having cataract were not affected by the presence of podoconiosis; adjusted OR 0.83 (95% CI 0.55–1.25), p = 0.36, and only significantly associated with age with an adjusted odds ratio of 1.07 (95% CI 1.05–1.09), p = 0.001 (Table 5).

**Table 5:** Clinical and socio-demographic correlates of any Cataract in East Gojam, Ethiopia.

Variables	Any Cataract n = 693^¶^		OR	P value	AOR*	P value
	Yes	No				
***Podoconiosis status***						
**Patient**	272	71	0.87 (0.59–1.26)	0.47	0.83 (0.55–1.25)	0.36
**Healthy control**	286	64	1		1	
***Age***			1.07 (1.05–1.09)	0.004	1.07 (1.05–1.09)	0.001
***Sex***						
**Male**	340	86	0.88 (0.60–1.31)	0.49	0.76 (0.50–1.16)	0.23
**Female**	218	49	1		1	
Wealth index**						
**Poor**	195	34	1.81 (1.10–3.00)	0.02	1.37 (0.80–2.33)	0.25
**Moderate**	184	54	1.06 (0.70–1.64)	0.80	0.87 (0.54–1.38)	0.55
**Wealthy**	179	47	1		1	
***Occupation***						
**Farmer**	547	126	3.28 (1.35–7.94)	0.01	2.93 (1.14–7.79)	0.31
**Other**	11	9	1		1	

*Binary logistic regression controlling for podoconiosis group, age, sex, occupation and socio-economic status.

** Calculated using Principal Components Analysis to reduce 15 wealth index variables down to three categories of socio-economic status.

^*¶*^ Seven participants could not have cataract examinations in either eye due to severe corneal opacities bilaterally.

### Trachomatous inflammation, scarring and corneal opacity between groups

Podoconiosis patients were found to have a significantly higher prevalence of trachomatous inflammation, trachomatous scarring and corneal opacity than healthy controls (p < 0.001 for each).

## Discussion

The study found that podoconiosis patients have worse visual acuity than healthy neighbourhood controls, with many more podoconiosis patients classified as blind. The prevalence of TT causing low vision through corneal opacification is higher in podoconiosis patients, creating a double burden of neglected tropical disease in this population. No significant difference in the prevalence of cataract was observed between podoconiosis patients and controls, however a higher number of podoconiosis cases had dense cataract (grade 4 or 5) and previous cataract surgery when compared to their neighbourhood controls.

NTDs commonly overlap within a population. Indeed, 80 million people in Ethiopia live in areas where one or more NTDs co-exist. Mapping of NTDs in Ethiopia has shown much geographical overlap between podoconiosis and other NTDs. For example Nationwide mapping has shown that 29 districts in Ethiopia are co-endemic for LF and podoconiosis, 116 co-endemic for onchocerciasis and podoconiosis, 302 co-endemic for trachoma and podoconiosis, 342 co-endemic for SHT and podoconiosis [37]. However, little is known regarding the overlap of NTDs within individuals. One study showed an overlap between Soil Transmitted Helminth (STH) infection and podoconiosis [38]. The authors concluded that this was likely to be a result of barefoot practices predisposing the individual to both diseases, rather than a shared biological mechanism. The association between the two NTDs podoconiosis and TT can be hypothesised to be the result of both shared environmental risk factors and a common biological pathology.

It is known that NTDs often co-exist within a population due to shared environmental risk factors such as sanitation, hygiene, poverty and access to health care [2,39]. The management of both trachoma and podoconiosis share common hygiene messages; for example, trachoma elimination programmes have focused on promoting facial cleanliness, while podoconiosis programmes educate patients about regular foot washing practices [41,16]. Both diseases are associated with reduced availability of water, sanitation and hygiene (WASH) facilities.^40^ Similarly, both diseases have been linked to poverty [42,14]. In this study podoconiosis patients were found to be significantly poorer than their neighbourhood controls, as we would expect from previous studies [41]. Likewise, trachoma is widely considered a disease of poverty [42].

Podoconiosis has a social impact and this in turn may lead to an increased burden of eye disease, independent of an association with trachoma [11]. Alongside economic poverty, individuals with podoconiosis are marginalised and stigmatised within their societies, leading to reduced living standards in comparison to their healthy neighbours [11,14]. Marginalisation within society alongside poor living standards among individuals living with podoconiosis may predispose to eye disease and reduced visual acuity through affecting health seeking behaviour [43], attendance for surgical procedures and disease prevention awareness.

Alongside an environmental and socio-economic hypothesis, it is possible that the association between podoconiosis and TT is also a result of shared biological pathology in these two chronic, scarring inflammatory diseases [44,45]. Previous research investigating pro-inflammatory and pro-fibrotic markers in serum and HLA associations in these two diseases could provide some insight into a potential shared pathology [8]., To date, very few studies have investigated the systemic effects of podoconiosis. Bilateral leg lymphoedema is thought to be caused by elements common in irritant volcanic soils (eg. aluminium, silicon, magnesium and iron) being absorbed through the foot and entering lower limb lymph nodes [46,7,10,47,48]. While it is the foot which facilitates dermal absorption, it is possible that these irritant elements cause wider systemic inflammatory effects. Addisu et al [44] compared levels of oxidative stress biomarkers in podoconiosis patients and healthy controls and found higher levels of these biomarkers in the serum of podoconiosis patients, suggesting inflammation in the early stage of the disease. Burton et al studied progressive trachomatous conjunctival scarring in an Ethiopian and Tanzanian cohort. They found that scarring progressed over a two year period in the absence of *C*. *trachomatis* [45]. Progressive scarring was associated with mucosal inflammation with an increased association in individuals with more frequent inflammatory episodes. This chronic conjunctival inflammation was associated with increased expression of pro-inflammatory factors and extracellular matrix regulators systemically including the pro-fibrotic factor CTGF, closely associated with TGFβ. Both podoconiosis and progressive trachomatous scarring have been associated with altered levels of pro-inflammatory factors in the serum. It is possible that systemic inflammation in podoconiosis patients could provide an initial hypothesis to explain the increased levels of trachomatous inflammation, trachomatous scarring and trachomatous trichiasis among podoconiosis cases. Further studies viewing podoconiosis as a systemic disease are required to investigate further potential effects of chronic systemic inflammation in these patients.

Many individuals who come into contact with irritant volcanic soils do not go on to develop podoconiosis [46,49]. Similarly, the natural history of trachoma varies significantly in prevalence and severity between families and communities with shared environmental risk factors [50]. The gene-environment interaction for both these diseases has been investigated, including the frequency of HLA antigens in both podoconiosis and blinding trachoma. Both podoconiosis and trachoma are associated with HLA Class II suggesting both are T cell mediated inflammatory diseases [8,51]. A shared pathogenesis associated with HLA Class II may account for some of the association between these two NTDs.

Both podoconiosis and TT have been shown to worsen poverty, reduce quality of life and increase the burden of disability in predominately poor rural communities [52,53,54,55,56]. Understanding that individuals can be burdened with both diseases could reduce the disease-specific approach to management and lead to an integrated management of these two NTDs at different levels. Firstly, the screening and diagnosis of these diseases could combine using the same community based health extension worker led household screening that is seen in trichiasis screening in Amhara region, Ethiopia. Both are highly visible diseases making community based screening very effective. Secondly, integrated treatment approaches could focus on common hygiene messages among the two groups and a focus on the improvement of WASH facilities, a key element of NTD management projects globally [40].

A key limitation of this study is the absence of a detailed slit lamp ophthalmic examination of the anterior segment and retina of the eye. While we found that TT is more common in this population, we were unable to study the prevalence of other eye diseases that could also account for the reduced vision in podoconiosis patients. Examining for cataract using direct ophthalmoscopy rather than slit lamp risks missing certain types of cataract, in particular nuclear cataract. A further limitation is the possibility of selection bias. While 460 podoconiosis patients were invited to take part, there was a high attrition rate before enrolment in the study with only 350 enrolling. Those with more disabling podoconiosis or worse vision may have been less likely to attend the local health facilities due to difficulties of travel. Lastly, the study only took place in one small rural population of northern Ethiopia reducing the generalizability of results to other populations affected by podoconiosis, trachoma and cataract.

## Conclusions

We conclude that podoconiosis patients have a greater burden of visual impairment than individuals living in the same neighbourhood without the disease. They are more likely to suffer from TT, and other stages of trachoma. Podoconiosis patients are poorer than their neighbours without the disease, but this alone may not be enough to account for this association and the significant difference in their burden of poor vision and blindness. Alongside shared environmental risk factors, shared biological mechanisms between these two NTDs, podoconiosis and TT, may contribute to the association that has been found and warrants further research to gain a better understanding of their co-endemicity. In particular, a focus in the future of studying and managing these two diseases together may help to reduce their burden in this northern Ethiopian population and farther afield.

## Supporting information

S1 Checklist STROBE checklist. (DOC)

S1 Data Data set cases and controls. (SAV)
